# Supplementary material for: Proteomic Analyses Detect Higher Expression of C-Type Lectins in Imidacloprid-Resistant Colorado Potato Beetle Leptinotarsa decemlineata Say
Source: Insects. 2020 Dec 23;12(1):3. doi: 10.3390/insects12010003 (PMC7822175; doi:10.3390/insects12010003)
Supplement: Supplementary file 1 [file insects-12-00003-s001.pdf]

Proteomic Analyses Detect Higher Expression of C-Type Lectins in Imidacloprid-Resistant Colorado Potato Beetle *Leptinotarsa decemlineata* Say

Ian M. Scott [1,*], Gabrielle Hatten [1], Yazel Tuncer [1], Victoria C. Clarke [2], Kristina Jurcic [2] and Ken K.-C. Yeung [2]

[1] London Research and Development Centre, Agriculture and Agri-Food Canada, London, ON N5V 4T3, Canada; ghatten@uwo.ca (G.H.); ytuncer@uwo.ca (Y.T.)
[2] London Regional Proteomics Centre, Biochemistry, Western University, London, ON N6A 5C1, Canada; vwraw@uwo.ca (V.C.C.); kjurcic@uwo.ca (K.J.); kyeung@uwo.ca (K.K.-C.Y.)
* Correspondence: ian.scott2@canada.ca

Supplementary Material

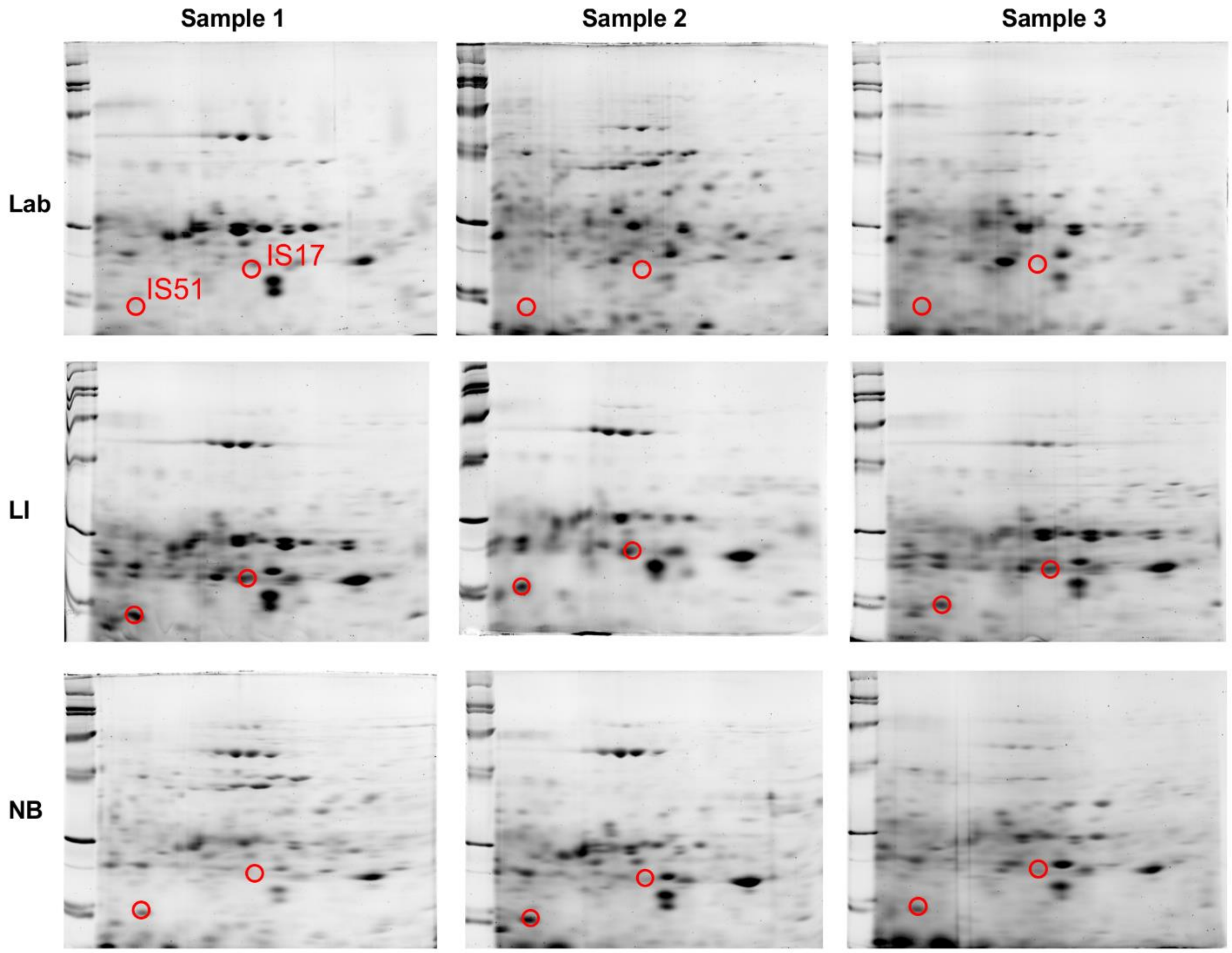

Figure S1. 2-D PAGE midgut samples from untreated Lab, LI and NB CPB strains. Strips 18 cm in length were run at pH 4-7 and cut to 7cm.

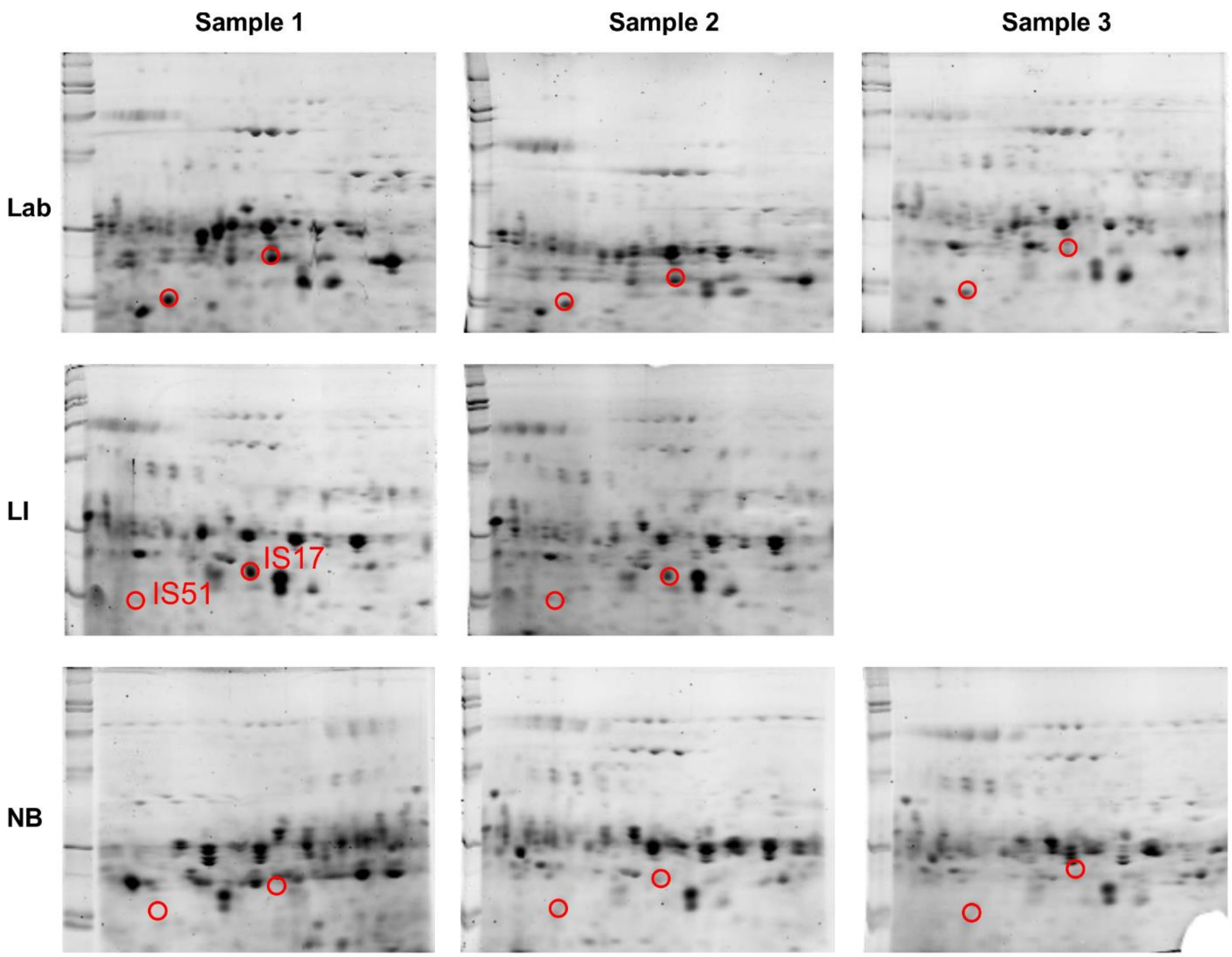

Figure S2. 2-D PAGE midgut samples from imidacloprid-treated Lab, LI and NB CPB strains. Strips 18 cm in length were run at pH 4-7 and cut to 7cm.

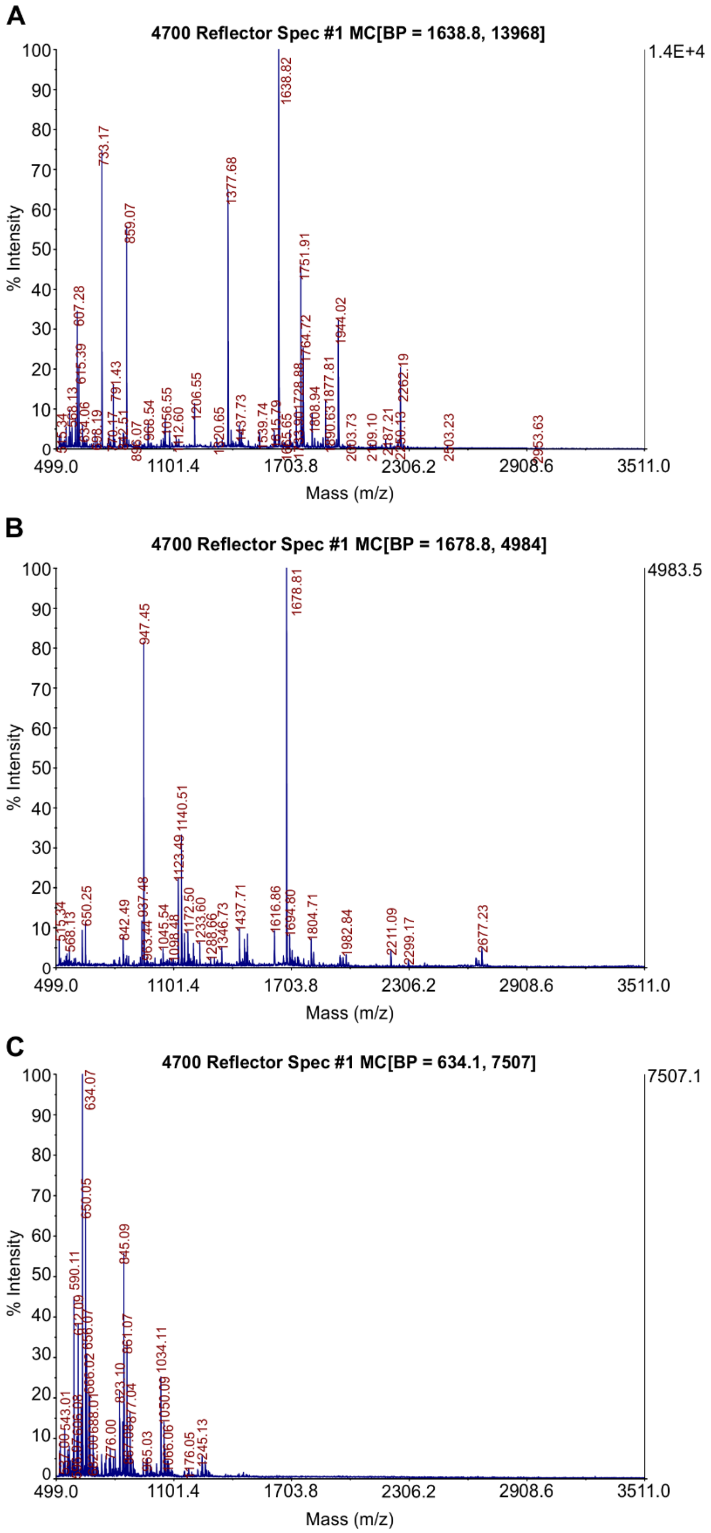

Figure S3. MALDI MS fingerprint spectrum for Lab strain (A), LI (B) and NB (C) IS17 spots.

Table S1. Summary of best hits for IS17 in Lab, LI and NB strain CPB midgut tissue[1] based on ESI LC-MSMS PEAKS output.

Sample	Hit #	MOWSE score	#Peptides #Match %Match	Percent coverage	Percent TIC	Protein MW (Da)/pI	Accession	Protein name
LI (69 peaks)	1	1490000	10/14/2010	40.6	48	19716/5.7	6198	gi|1285030099|ref|XP_023027360.1 | ladderlectin-like [*L. decemlineata*]
	2	175212	12/17/2012	4.8	19.6	250525/5.2	8461	gi|1285025224|ref|XP_023024904.1 | dynein beta chain, ciliary-like [*L. decemlineata*]
	3	37519	17/15/22	4.5	41.3	488975/5.8	9002	gi|1285024081|ref|XP_023024309.1 | dynein heavy chain 10, axonemal [*L. decemlineata*]
Lab	1	111000000	44/32/36	6.6	40.2	778404/5.4	8376	gi|1285025405|ref|XP_023028950.1 | uncharacterized protein LOC111517131, partial [*L. decemlineata*]
	2	53100000	25/22/25	6.5	10.1	481588/6.0	7163	gi|1285028007|ref|XP_023026282.1 | cytoplasmic dynein 2 heavy chain 1 [*L. decemlineata*]
	3	144256	17/16/18	7	9.7	287608/8.6	11198	gi|1285019357|ref|XP_023021916.1 | protein 4.1 homolog isoform X3 [*L. decemlineata*]
NB	1[2]							

[1]Digestion type = trypsin for all samples; [2]MS-Fit could not fit the data to any proteins.
